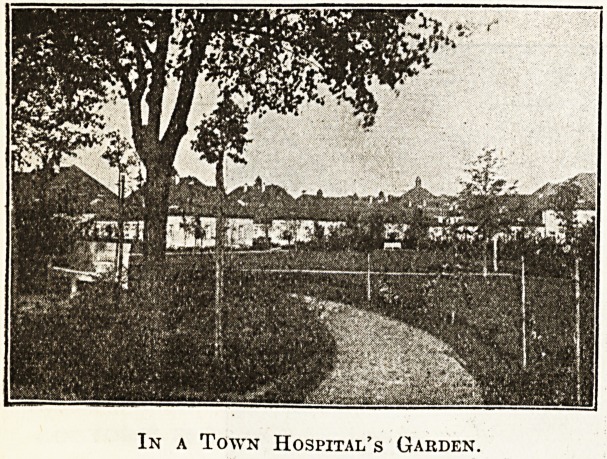# Government (Teaching) and Municipal (Non-Teaching) German Hospitals


**Published:** 1912-10-26

**Authors:** Henry Burdett


### GOVERNMENT (Teaching) and MUNICIPAL (Non-Teaching) GERMAN HOSPITALS.*

By SIR HENRY BURDETT, K.C.B.

### III. No Free but all Pay Patients.

In our article last week under this title we omitted to point out that the hospital authorities in Germany can at any time readily prevent any such propaganda as that referred to. The way is simple. The hospital authorities alone know who number So-and-so is, and what class and which type of person each patient is. It rests with them, therefore, by classifying the pavilions for administrative purposes, to free the respectable artisans and other members of the community from any elements which might otherwise prove objectionable or even worse. Classification has effected immense good in our Poor-Law infirmaries. Indeed, experience proves that the absence of such classification sometimes, as in the maternity branch of a great establishment like the Brownlow Hill Infirmary, Liverpool, inflicts needless misery on innocent patients.

Ward Blocks, Gardens and Avenue.
(Note.—This early block does not do justice to the gardens of to-day.)

### Paying Patients Proper.

In the German hospitals every patient is paid for. The accommodation provided for patients in the various classes is as follows: The first or ordinary class, where everyone is charged the minimum rate of 2½ to 3½ marks per diem. This class of patient is accommodated in a general ward containing from 18 to 20 beds, and is provided with everything that the circumstances of his case may require or his necessities may demand. The payments made on behalf of this class may or may not be sufficient to defray the whole cost of the patients' treatment and maintenance when in the hospital. If they are not sufficient, then, in Kasse or approved society cases, the State or municipality at the end of each year makes up the deficiency represented by the difference between the actual expenditure on Kasse cases and the actual payments received from the Kasse for the maintenance of such patients, and advances the difference so as to balance the hospital's accounts for the year. Any such difference paid has to be made good to the State or municipality by the Kasse, which increases the amount of its levies upon its members in the ensuing year and so recoups these authorities the actual sum which was over-expended on the Kasse patients' behalf in the past twelve months.

### Higher Class Patients.

In the second class of cases, where the patient is placed in a ward with two beds, the price charged per diem is from 3 to 5 marks. The third class of patients pay from 7 to 10 marks per diem as a minimum. (All these fees are in addition to extras of various kinds.) In class 3 each patient is placed in a single room which he has to himself. These rooms contain a second bed where the patient may have a friend or a special nurse, for which, of course, extra payment is made. In this class (3), if a patient wishes to have a special nurse the payment for her food at Nüremberg, for instance, is 4 marks extra per diem. In classes 2 and 3 private patients are treated generally in a special building and in well-furnished private rooms with special food. Each patient's room contains a tariff of charges and a menu, so that every patient can order, subject to the control of the doctors, what food he requires. It may be well to add that patients in the first or lowest class are supplied with complete sets of clothing and every requisite for use during their residence. The patients of the second and third classes, who pay higher fees, provide their own clothing, cutlery, and personal equipment.

* The previous articles in this series appeared in our issues of October 12 and 19.

### Special Fees and Extras.

In addition to the charges per diem just recited, classes 2 and 3 are liable to pay the following extras: Wine, mineral waters, and beer at wholesale prices, plus 10 per cent. as a security against breakages and other losses. The fees paid for operations and special medical attendance by the private patients are fixed for each case by the director of the hospital. A special scale of fees is instituted for Röntgen and Light treatment, for medicinal and other baths, for massage, and for a number of other things which we shall deal with later on. There is usually a special fee for children, who are generally only admitted in exceptional cases or cases of extreme need, and are paid for at the rate of 1 mark per diem for a child up to 3 years of age and 2 marks per diem for children from 4 to 13. The Stadt hospitals in different cities have different regulations, but it is often the rule to confer the right of hospital treatment in a Stadt hospital upon the residents or ratepayers in the particular city. In the same way members of friendly societies who can claim hospital treatment are admitted under class 1, and they are paid for at that rate by the Kasse or approved society to which they belong. But when any such patient so desires he may be admitted to the pay pavilion, providing he agrees to pay the difference per diem between the Kasse rates and those of the second or third class he selects to enter. Extras are usually paid for in cash when they are incurred. In some of the Stadt hospitals, but by no means in all, a deposit is required on the admission of a patient, as, for instance, at Munich, where the rule is that each patient shall pay or provide on admission as a deposit or guarantee as follows: A private patient in a special room, first class, deposits at least 100 marks; a private patient of the second or third class deposits 70 marks; in the case of members of a Kasse or friendly society the deposit amounts to 45 marks.

In a Town Hospital's Garden.

In those hospitals in Germany where no deposits are made we were assured that there were no bad debts incurred by the hospitals, and that no credit was given; each patient in every class, or his friends, paid to the hospital every week the whole cost incurred during his last seven days' residence there. In the case of approved societies, however, several hospitals arrange that these societies shall pay monthly the whole of the money due to the hospital for the treatment of their members.

### The Evolution of Pay Patients' Pavilions.

It will be seen from the information we have given about the fees paid and the accommodation provided for paying patients of the higher classes that the accommodation in the better class hospitals is of the best. If we are to judge from the attitude of mind of the authorities who were responsible for the plans of the earlier Stadt hospitals, and recently by the actual condition and position on the site of these pay pavilions, we should be forced to the conclusion that twenty years ago the higher grades of pay patients were not specially welcome, or at any rate were not expected to be numerous. As years and experience have accumulated the pavilions for pay patients have extended and the accommodation has improved. The most noticeable improvement is the recognition that patients who pay well for hospital accommodation should have the best that money can provide. In the earlier hospitals some pay pavilions are not attractive. Amongst the causes of the defects has been the situation of this pay pavilion on the site, where it is sometimes overshadowed by other and larger buildings, and where there seems to have been too little attempt to provide the maximum of air and light for the benefit of the occupants. We formed the conclusion that the pay pavilion has, in fact, supplied a distinct and growing need of the middle and better classes in Germany. This is shown by the improvement in the nature of the accommodation provided in the newest hospitals in Munich, and especially by the situation of the paying patients' pavilions on the site of the most recent Stadt hospital, that at Bambeck, Hamburg. In the plans of this hospital the paying pavilions occupy a position of honour. They take the foremost place and occupy probably a better position on the site than any other department of the hospital. They are placed where in other days the church or chapel would have been placed. The reason for this distinction is, we fancy, that the higher classes of pay patients are becoming a more valuable asset in the hospital economy.

It is thirty-six years ago since the question of pay patients and the need of hospital accommodation of this type was strongly insisted upon throughout the newspaper press of Great Britain. The result of that agitation was the erection in Fitzroy Square of the first Home, or pay hospital, in the British Islands, an institution which was rebuilt about nine years ago. To-day it is still one of the most popular and useful of hospitals. It has for thirty years paid its way. During these years periodical attempts have been made to introduce into the British system of voluntary hospitals paying pavilions. Many of the voluntary hospitals have sites available upon which such buildings could be erected. We hope that one result of the reorganisation of hospital relief upon which our hospital system rests will be the speedy erection of paying pavilions, by the authorities of the voluntary hospitals, to the increase of their usefulness, and the great improvement of their financial position.

*(To be continued.)*

## Figures and Tables

**Figure f1:**
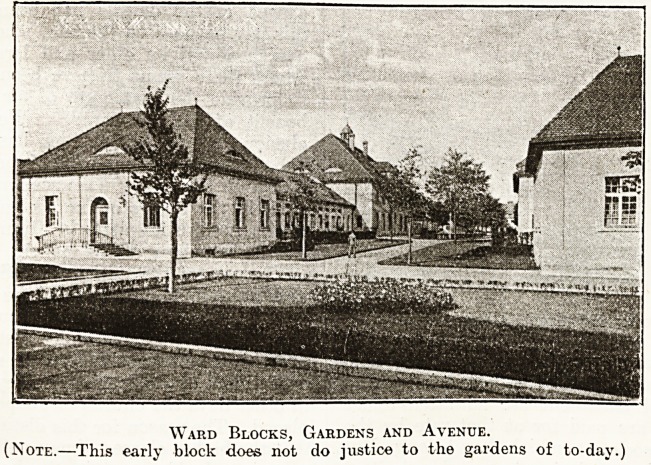


**Figure f2:**